# Possibility of graphene growth by close space sublimation

**DOI:** 10.1186/1556-276X-9-182

**Published:** 2014-04-14

**Authors:** Mykola V Sopinskyy, Viktoriya S Khomchenko, Viktor V Strelchuk, Andrii S Nikolenko, Genadiy P Olchovyk, Volodymyr V Vishnyak, Viktor V Stonis

**Affiliations:** 1 V. Ye. Lashkaryov Institute of Semiconductor Physics, National Academy of Sciences of Ukraine, 45 Prospect Nauky, Kyiv 03028, Ukraine; 2 G. V. Kurdyumov Institute for Metal Physics, National Academy of Sciences of Ukraine, 36 Vernadsky Blvd, Kyiv 03680, Ukraine

**Keywords:** Graphene films, Deposition, Graphite, Sublimation, Si/SiO_2_ substrate, Raman spectroscopy, Ellipsometry, 68.65.Pq, 81.15.-z, 81.05.ue

## Abstract

Carbon films on the Si/SiO_2_ substrate are fabricated using modified method of close space sublimation at atmospheric pressure. The film properties have been characterized by micro-Raman and X-ray photoelectron spectroscopy and monochromatic ellipsometry methods. Ellipsometrical measurements demonstrated an increase of the silicon oxide film thickness in the course of manufacturing process. The XPS survey spectra of the as-prepared samples indicate that the main elements in the near-surface region are carbon, silicon, and oxygen. The narrow-scan spectra of C1*s*, Si2*p*, O1*s* regions indicate that silicon and oxygen are mainly in the SiO_*x*_ (*x* ≈ 2) oxide form, whereas the main component of C1*s* spectrum at 284.4 eV comes from the *sp*^2^-hybridized carbon phase. Micro-Raman spectra confirmed the formation of graphene films with the number of layers that depended on the distance between the graphite source and substrate.

## Background

Graphene attracts enormous interest due to its unique properties, such as high charge carrier mobility and optical transparency, in addition to flexibility, high mechanical strength, environmental stability [1-3]. These properties have already had a huge impact on fundamental science and are making graphene and graphene-based materials very promising for the whole series of applications starting with electronics and ending with medicine [2,3]. It should be noted that currently the studies dealing with graphene are not limited to single-layer samples; the structures containing two or more graphene layers are also of interest [4].

In addition to deepening the understanding of the fundamental aspects of this material, the present stage of graphene research should target applications and manufacturing processes. Large-scale and cost-effective production methods are required with the balance between ease of fabrication and materials' quality [2,3]. The placement of graphene on arbitrary substrates is also of key importance to its applications. The ideal approach would be to directly grow graphene where required (including dielectric surfaces). Despite the fact that at present there are quite a few proposed methods for the preparation of graphene films, we are still far from these goals [3]. Therefore, further development of the existing methods of graphene film production as well as invention of new ones is in order.

Our first attempts to deposit graphene films directly onto the Si-SiO_2_ substrate should be considered in view of the abovementioned requirements. The close space sublimation (CSS) technique is very attractive in this sense because it is simple, inexpensive, and can be adapted for industrial use. Here we report our research into growing graphene films using CSS at atmospheric pressure.

## Methods

The CSS technique was first proposed 50 years ago [5]. It is based on the thermal-heating-induced sublimation of the source's material followed by vapor condensation onto the closely spaced substrate. The cross dimensions of the source and the substrate greatly exceed the distance between the source and the substrate. So far the CSS technique has been widely used in the production of thin films for solar cell applications [6]. To our knowledge, CSS has not yet been used for production of graphene films. We simplified the design of the setup intended for film deposition as much as possible. In our case, carbon films were deposited using the thermal sublimation of the graphite at atmospheric pressure in the quasi-closed volume created inside a muffle furnace. This volume was the fused quartz crucible with ground stopper filled with densely packed fine TiO_2_ powder. (TiO_2_ was used because of its good chemically stability, high temperature stability, and corrosion resistance). Such a design has ensured reproducible results. The growth temperature was 850°C. The substrate was 130-nm-thick SiO_2_ film on silicon wafer obtained by oxidizing it in air at 1,100°C. Two types of film were investigated: one obtained using direct contact between the graphite plate and substrate (type I) and another obtained at 300-μm distance (type II).

Raman spectroscopy is one of the most effective tools for characterization of *sp*^2^ nanostructures, including graphene films. Specifically, the shape of the 2D Raman peak may serve as the fingerprint to distinguish single-, bi- and few-layer graphenes [7]. That is why initially the prepared samples have been investigated by Raman spectroscopy. X-ray photoelectron spectroscopy (XPS) and ellipsometry are among the most powerful tools for investigation of very thin films. This determined the choice of these methods for the characterization of the obtained carbon deposits.

Micro-Raman spectra in the 1,000 to 3,000 cm^-1^ spectral range at room temperature and excitation wavelength 488 nm were registered using Horiba Jobin-Yvon T-64000 Raman spectrometer (Horiba Ltd., Edison, Kyoto, Japan). The laser spot size at the focus was around 1 μm in diameter, and laser power at the sample was 1 mW. The laser power density used (approximately 1 mW/μm^2^) was the maximum at which the heating of the sample there was not observed yet (i.e., at which there was no observable temperature shift of the phonon bands). Spectral resolution was 0.15 cm^-1^.

XPS was obtained on JSPM-4610 photoelectron spectrometer with Mg K_*α*_ (1,253.6 eV) X-ray source. The film deposition process was analyzed by monochromatic multi-angle ellipsometry (*λ* = 632.8 nm) using LEF-3 M-1 laser null ellipsometer and in-house-developed software modeling optical characteristics of thin-film structures (birefringence, dichroism, uniformity over depth) [8]. The determination of quantitative values for the parameters that characterize these properties was achieved by solving the inverse task of ellipsometry (ITE): the true values of models' parameters were assumed to be the ones minimizing the mean-squared error (MSE):

$$\mathrm{MSE}=\sum \left[\;{\left\{\Psi^{\exp}\left({\varphi_{0}}_{i}\right)–\Psi^{\mathrm{mod}}\left({\varphi_{0}}_{i}\right)\right\}}^{2}+{\left\{\Delta^{\exp}\left({\varphi_{0}}_{i}\right)–\Delta^{\mathrm{mod}}\left({\varphi_{0}}_{i}\right)\right\}}^{2}\right],$$

where *Ψ*^exp^(*φ*_0*i*_), Δ^exp^(*φ*_0*i*_) are experimentally measured values of polarization angles *Ψ*, Δ for 13 incidence angles *φ*_0*i*_ from the range of 45° to 75° and *Ψ*^mod^(*φ*_0*i*_), Δ^mod^(*φ*_0*i*_) are calculated for the same incidence angles using the adopted model. As it will be seen below, in this study, it was sufficient to use single-layer and two-layer models with the following types of layers:

– Isotropic uniform transparent layer (IUTL) with *n*, *h*

– Isotropic uniform absorbing layer (IUAL) with *n*, *k*, *h*

– Unaxially anisotropic uniform transparent layer (UAUTL) with *n*_o_, *n*_e_, *h*

– Isotropic linearly non-uniform transparent layer (ILNUTL) with *n*_b_, *n*_t_, *h*

– Isotropic linearly non-uniform absorbing layer (ILNUAL) with *n*_b_, *n*_t_, *k*_b_, *k*_t_, *h*

Here, *h* is the layer thickness and *n, k* are refractive and absorption index, respectively. Lower subscripts denote the following: o, ordinary; e, extraordinary; b, bottom; t, top.

The measured area was approximately 1 μm^2^ for micro-Raman, approximately 1 mm^2^ for ellipsometric, and approximately 20 mm^2^ for XPS measurements.

## Results and discussion

**Figure 1:**
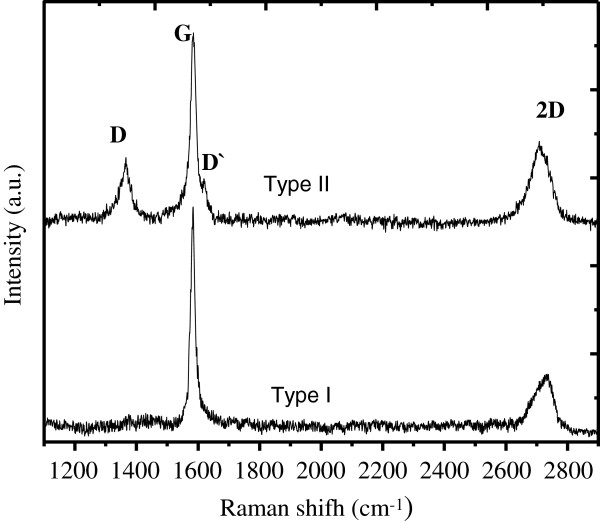
Micro-Raman spectra measured on the samples of type I and type II.

Micro-Raman spectra in most of the measured points of the sample of type II were weak in intensity as well as unstructured. However, on the sample, there are local areas where the spectra are more intense and structured. One of them is shown on Figure 1 (upper curve). As a rule, micro-Raman spectra measured in various regions of the type I sample are more intense as compared to the type II sample spectra. They correspond to the Raman spectra of the graphite-like carbon phase with various degrees of order - D band is present in some of them and is absent in some others. One of the spectra without D band is also presented on Figure 1 (lower curve). As can be seen, in the spectra measured in more ordered regions of both types of samples, the G band is narrow (half-width ≤20 cm^-1^). This indicates the formation of non-amorphous *sp*^2^ carbon phase in these regions.

More detailed information about the structure of *sp*^2^ carbon phase can be obtained from the 2D band analysis. Both the position and the shape of this band are different in these two spectra. The 2D band in both spectra is asymmetric. However, the details of this asymmetry differ.

**Figure 2:**
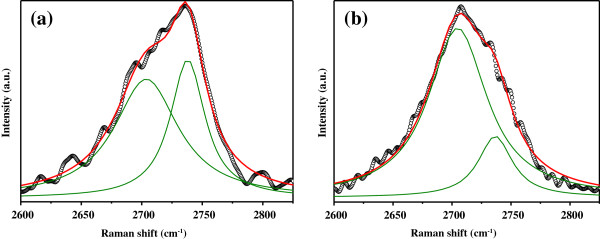
**Enlarged 2D band regions of micro-Raman spectra measured on samples.** Type I **(a)** and type II **(b)**. Open circles are the experimental data, while the green and red curves indicate the fittings of the experimental data by Lorentzian functions. The fitting peaks and peak sum are shown by the green and red curves, respectively.

In type I sample, the band has the maximum at 2,732 cm^-1^ with a gentler drop on the low-energy side. This kind of asymmetry is inherent to graphite with AB layer packing and to the multilayer graphene with the same type of packing. In Figure 2a, the 2D band of type I sample is presented on a larger scale. Detailed visual examination of this band shows great similarity of its shape and position to those for the 2D band of mechanically cleaved six- to seven-layer graphene films on SiO_2_/Si substrate [9]. As can be seen from Figure 2a, the 2D band is rather well fitted by two Lorentzian components (2D_1_, 2D_2_). The deconvolution of the band confirms the foregoing visual observation: the quantitative values of the parameters of the subbands 2D_1_ and 2D_2_ are closer to the several layer graphene than to graphite - the distance between the subbands is approximately 33 cm^-1^, which is closer to the 26 cm^-1^ value calculated for the six-layer graphene [7] than to the 44 cm^-1^ value for HOPG.

In the type II sample, the band has the maximum at 2,709 cm^-1^ with the gentler drop on the high-energy side. The enlarged 2D band region of the type II sample is shown on Figure 2b. A detailed visual examination of this band shows that its shape and position are analogous to those observed for graphene films with number of layers 2 ≤ *n* ≤ 4 [10-12]. From Figure 2b, it is also seen that the experimental 2D band is well fitted by two Lorentzian components. The characteristics of the deconvolution are similar to the characteristics of the 2D band deconvolution for micromechanically cleaved three- to four-layer graphene sheets on SiO_2_/Si substrate [12]. There is yet another indication that the type II sample film has fewer graphene layers as compared to the type I sample - despite the greater number of defects in the type II sample (confirmed by the presence of D band in its spectrum), the *I*_2D_/*I*_G_ ratio in the type II sample is still greater than in the type I sample.

Since the type II sample had fewer graphene layers, it had been studied in greater detail using XPS and ellipsometrical methods.

**Figure 3:**
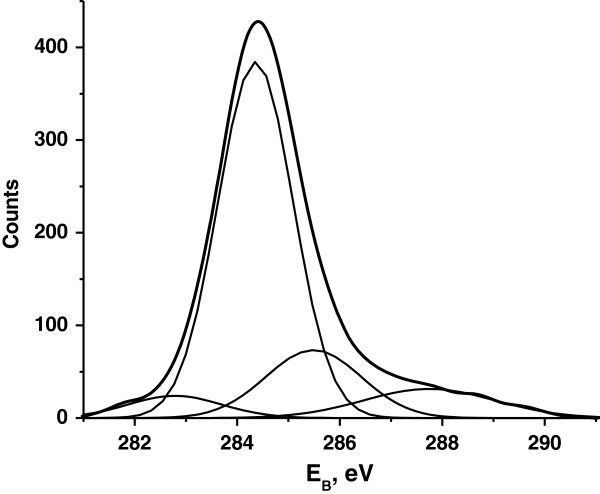
**C1*s *XPS spectrum of the type II sample.** The thick curve is the original data. The thin curves are the fitting peaks on 282.8, 284.4, 285.5, and 287.8 eV. The summary fitting curve almost completely matches the experimental curve.

The XPS survey spectrum (0 to 1,000 eV) of the type II sample shows that the main elements in the near-surface region are carbon, silicon, and oxygen. The narrow-scan (step 0.05 eV) XPS spectra of Si2*p*, O1*s* core levels (not presented here) indicate that silicon and oxygen are mainly in SiO_*x*_ (*x* ≈ 2) oxide form. The C1*s* core level narrow-scan XPS peak is asymmetrical, and four components are required to achieve the accurate fit to the data (Figure 3). The largest contribution at 284.4 eV comes from the *sp*^2^-hybridized carbon phase. Other weak contributions can be attributed to the following: 282.8 eV - *sp*^1^ carbon atoms or Si-C bonds, 285.5 eV - *sp*^3^ carbon atoms and/or C-O, C-OH bonds, and 287.8 eV - carbonyl groups [13-15]. Comparison of the intensities of C1*s*, Si2*p*, O1*s* peaks demonstrates that the overall (*brutto*) composition of near-surface region is close to ‘С_1_Si_1_O_2_’. Such quantitative relation of these three elements is possible under one of the following scenarios: (a) the surface of the SiO_2_ film is covered by a compact, approximately 1-nm-thick carbon layer; (b) the surface of the SiO_2_ film is covered by thicker but non-compact carbon layer; (c) the near-surface region of the sample consists of silicon dioxide film with carbon inclusions (SiO_2_ < C>). Various simultaneous combinations of these three cases cannot be excluded.

The fitting of experimental angular dependences *Ψ*(*φ*_0_), Δ(*φ*_0_) for the initially oxidized silicon substrate in terms of two-parameter IUTL-model produced a sufficiently small value of the error function (MSE_min_ = 0.1434) for the values of variable parameters *n* = 1.460, *h* = 135.7 nm (the values of the optical constants of the silicon substrate here and in the rest of the calculations are *n*_s_ = 3.865, *k*_s_ = 0.023). In terms of IUTL-model, *n* and *h* can, in fact, be calculated from the values of Ψ and Δ measured at any given *φ*_0_. Values of *n* and *h* obtained this way fluctuate randomly in the ranges of 1.459–1.461 and 135.5 nm – 135.8 nm when *φ*_0_ changes from 45° to 75°. In this case, the absence of clear dependence of *n* and *h* from *φ*_0_ suggests the IUTL model's adequacy as a necessary condition had been met. Minimization of MSE in terms of the three-parametric single-layer models that allow individual evaluation of the absorption, anisotropy, and refractive index vertical non-uniformity does not decrease the value of MSE_min_ - these models, in fact, get reduced to IUTL model:

$${\mathrm{MSE}}_{\min}=0.1305\;\mathrm{at}\;n=1.458,\;k=0.001,\;h=136.1\;\mathrm{nm}$$

$${\mathrm{MSE}}_{\min}=0.1426\;\mathrm{at}\;n_{o}=1.4602,n_{e}=1.4599,h=135.7\;\mathrm{nm}$$

$${\mathrm{MSE}}_{\min}=0.1426\;\mathrm{at}\;n_{b}=1.4614,n_{t}=1.4590,h=135.8\;\mathrm{nm}$$

This should be considered as sufficient condition for IUTL-model adequacy. Thus, the oxide film obtained by oxidation of silicon on air is isotropic, uniform, and transparent. We emphasize that the *n* = 1.460 value corresponds to the refractive index value for SiO_2_ thermal oxide films.

Carrying out the graphite sublimation process leads to considerable changes of the *Ψ* - Δ values. These changes are accompanied by the decrease in adequacy of the IUTL model - there is observed monotonic increases of *n*(*φ*_0_) values from 1.457 to 1.466 and decrease of *h*(*φ*_0_) values from 151.7 to 150.4 nm as *φ*_0_ increases from 45° to 75°. This decrease in adequacy is also confirmed by computation of the MSE_min_ in the terms of IUTL-model - the MSE_min_ value increases by an order of magnitude:

$${\mathrm{MSE}}_{\min}=1.51\;\mathrm{at}\;n=1.462,h=150.8\;\mathrm{nm}.$$

As it can be seen within the framework of the IUTL-model, there is little change of *n* value, yet there is substantial increase of *h* value. This result shows that as far as the sample's optical properties are concerned, the most substantial result of carrying out the graphite sublimation process has been the thickening of the oxide film.

The reasons of the decrease in IUTL model adequacy can, in first approximation, be evaluated through solving of ITE in terms of three-parametric single-layer models. The results are as follows:

$${\mathrm{MSE}}_{\min}=0.72\;\mathrm{at}\;n=1.448,k=0.005,h=153.7\;\mathrm{nm}$$

$$\begin{array}{l} {\mathrm{MSE}}_{\min}=0.99\;\mathrm{at}\;n_{b}=1.478,n_{t}=1.448,\\ \;h=152.0\;\mathrm{nm}\;\left(n_{\mathrm{av}}=1.463\right) \end{array}$$

$$\begin{array}{l} {\mathrm{MSE}}_{\min}=1.484\;\mathrm{at}\;n_{o}=1.462,n_{e}=1.464,\\ \;h=150.75\;\mathrm{nm}\;\left(n_{\mathrm{av}}=1.463\right) \end{array}$$

As it follows from these solutions, the anisotropy is the least responsible factor for the deviation of the sample's optical properties from the IUTL model; to a greater extent, this deviation is due to the appearance of absorption as well as vertical non-uniformity.

The solving of ITE in terms of the five-parametric models that takes into account the presence in the sample of both absorption and non-uniformity (sharp or smooth) showed the more adequate character of the model with sharp non-uniformity:

$$\begin{array}{l} {\mathrm{MSE}}_{\min}=0.327\;\mathrm{at}\;n_{b}=1.462,n_{t}=1.449,k_{b}=0.013,\;\\ k_{t}=-0.001,h=153.0\;\mathrm{нм},\left(n_{\mathrm{av}}=1.451,k_{\mathrm{av}}=0.006\right),\\ {\mathrm{MSE}}_{\min}=0.221\;\mathrm{at}\;n_{l}=1.423,\;n_{u}=3.24,\;k_{u}=0.463,\;\\ h_{d}=149.5\;\mathrm{нм},h_{u}=1.0\;\mathrm{nm}. \end{array}$$

Lower subscripts denote the following: l, lower; u, upper. Note that in terms of both of these models, the *n* value of oxide film is below 1.46. It may be due to the appearance of porosity in the oxide film and/or change of its composition through the partial replacement of silicon atoms by carbon atoms.

The complication of the two-layer model by introducing birefringence, dichroism, non-uniformity in both lower and upper layers did not lead to any noticeable reduction of MSE_min_, despite the fact that the number of variable parameters increased to 8. The obtained values of the parameters describing the deviation of these models from the ‘lower IUTL - upper IUAL’ model were small in this case. This indicates the sufficient adequacy of the ‘lower IUTL - upper IUAL’ model. Let us turn to the values of the optical constants of thin upper film. Its refractive index value (3.24) is higher and absorption index value (0.463) is lower than the reported values for bulk graphite, the film consisting of 8 to 9 graphene layers, and single-layer graphene (*n* = 2.73, *k* = 1.42 are found at *λ* = 633 nm for bulk graphite [16]; *n* = 2.68, *k* = 1.24 at *λ* = 633 nm are found for the film consisting of 8 to 9 layers of graphene [17]; *n* = 2.7 to 2.8, *k* = 1.4 to 1.6 [18] and *n* = 2.5 to 2.7, *k* = 1.1 to 1.4 [19] have been reported for single-layer graphene). On the other hand, these values are very close to the values of the optical constants for *a*-C films deposited using pulsed laser deposition (*n* ~ 3.10, *k* ~ 0.40 at *λ* = 633 nm) [20]. Also, the value of *Imϵ* = 2 × 3.24 × 0.463 = 3.00 calculated based upon our data is in the middle of the range for the values *Imϵ* = 2.0 to 4.0. This range has been previously obtained at λ = 633 nm for laser-irradiated carbon films with a large amount of graphite phase and dominating *sp*^2^-type bonds [21].

Thus, from the ellipsometric analysis, it follows that as a whole, the upper film can be treated as a disordered graphite-like layer having the thickness approximately equal to three-layer graphene. This result proves the realization of the first scenario among those that are compatible with XPS measurements*.* Weak intensity as well as unstructured micro-Raman spectra in most of the measured points of the type II sample indicates the formation of the strongly disordered amorphous carbon-based phase with large number of defects. (Similar character of the Raman spectra had been observed, for example, in the carbon films obtained by the electron-beam-induced high-speed evaporation of graphite on substrates preheated to 700°C to 800°C [22]). The structure of such a type leads to significant (order of magnitude) decrease in inelastic light scattering cross-section. This, together with the small thickness of the film, explains the low intensity of the Raman signal in our case.

Thus, based on the data of all three characterization methods, we can state that in the sample of type II, the SiO_2_ film is covered with approximately 1-nm-thick film consisting of *sp*^2^ carbon-based highly disordered amorphous phase with some number of three-layer defective graphene inclusions.

Possible reasons for greater disordering and the number of defects of the in the type II sample deposited carbon film as compared to the type I one can be the greater distance between the source and substrate as well as a lot more gases of air in the sandwich during the type II sample preparation. Substantial changes in the silicon oxide film indicate the significant impact of the atmosphere taking place during the fabrication of the type II sample. First, its thickness increased, and its refractive index decreased. Second, attention should be given to the silicon oxide film growth rate during the graphite sublimation process: the oxide thickness increase was 13.4 nm in type II sample, but only 4.0 nm in the control Si-SiO_2_ sample placed in the oven near the quartz box. Such difference in the silicon oxidation rate can be explained by increase in the ‘source-substrate’ sandwich temperature. The increase in local temperature inside the sandwich is possible because the heating of graphite to 850°C in air should cause exothermic oxidation reactions with oxygen and water molecules [23]. Authors [24] showed that exposure of a few layer graphene films in air at *T* ≥ 600°C leads to the formation of defects. The defects are initially *sp*^3^ type and become vacancy-like at higher temperature [24]. Thus, the abovementioned facts make it possible to think that more defective structure of carbon deposit in the type II sample is to great extent caused by the greater amount of the active air gases (oxygen, water vapor) as well as the higher local temperature in the sandwich. All of this is the consequence of greater distance between the graphite plate and the substrate.

## Conclusions

The possibility of graphene fabrication using the simple and low-cost modified method of close space sublimation at the atmospheric pressure has been demonstrated. When carrying out carbon deposition under the same conditions, the thickness of several-layer graphene film decreases and its defectiveness increases with increase in the distance between the source and the substrate. This motivates further in-depth study of the mechanism of the film formation in order to develop the technological regimes that would allow fabrication of the better graphene films. First of all, it would be necessary to determine the influence of the atmosphere on the graphene film deposition process.

## Abbreviations

CSS: Close space sublimation; HOPG: Highly ordered pyrolytic graphite; ILNUAL: Isotropic linearly non-uniform absorbing layer; ILNUTL: Isotropic linearly non-uniform transparent layer; ITE: Inverse task of ellipsometry; IUAL: Isotropic uniform absorbing layer; IUTL: Isotropic uniform transparent layer; MSE: Mean-squared error; UAUTL: Unaxially anisotropic uniform transparent layer; XPS: X-ray photoelectron spectroscopy.

## Competing interests

The authors declare that they have no competing interests.

## Authors’ contributions

The idea of the study was conceived by VSK and MVS. VSK designed the deposition setup and conducted the growth of the films. VVS and ASN performed micro-Raman characterization. GPO conducted the ellipsometry measurements. VVV and VVS carried out XPS experiments. MVS interpreted the experiments and wrote this manuscript. All authors read and approved the final manuscript.
